# Supplementary material for: Investigating the Functional Role of the Cysteine Residue in Dehydrin from the Arctic Mouse-Ear Chickweed Cerastium arcticum
Source: Molecules. 2022 May 4;27(9):2934. doi: 10.3390/molecules27092934 (PMC9102250; doi:10.3390/molecules27092934)
Supplement: Supplementary file 1 [file molecules-27-02934-s001.zip › molecules-1667674-supplementary.pdf]

Supplementary Materials

Investigating the Functional Role of the Cysteine Residue in Dehydrin from the Arctic Mouse-Ear Chickweed *Cerastium arcticum*

Il-Sup Kim [1,†], Woong Choi [2,†], Ae Kyung Park [3], Hyun Kim [2], Jonghyeon Son [2,4], Jun Hyuck Lee [2,5], Seung Chul Shin [2], T. Doohun Kim [6] and Han-Woo Kim [2,5,*]

Table S1. Polymerase chain reaction (PCR) primers used in this study.

Primer	Nucleotide Sequence	Restriction Site
pET30a/WT(F)	CTGATATCGGATCC**<u>GAATTC</u>**ATGTCGAACTTACACCCAAC	*Eco*RI
pET30a/WT(R)	TGGTGGTGGTGGTG**<u>CTCGAG</u>**TTAGTAACCCTTGTGAGCTT	*Xho*I
pGPD426/WT(F)	CCCCCGGGCTGCAG**<u>GAATTC</u>**ATGTCGAACTTACACCCAAC	*Eco*RI
pGPD426/WT(R)	AACTAATTACATGA**<u>CTCGAG</u>**TTAGTAACCCTTGTGAGCTT	*Xho*I
C143A(F)	CCTACCGGTGACCACGCCCACCAGGAGCAG	-
C143A(R)	CTGCTCCTGGTGGGCGTGGTCACCGGTAGG	-

F, forward primer; R, reverse primer; WT, wild type.

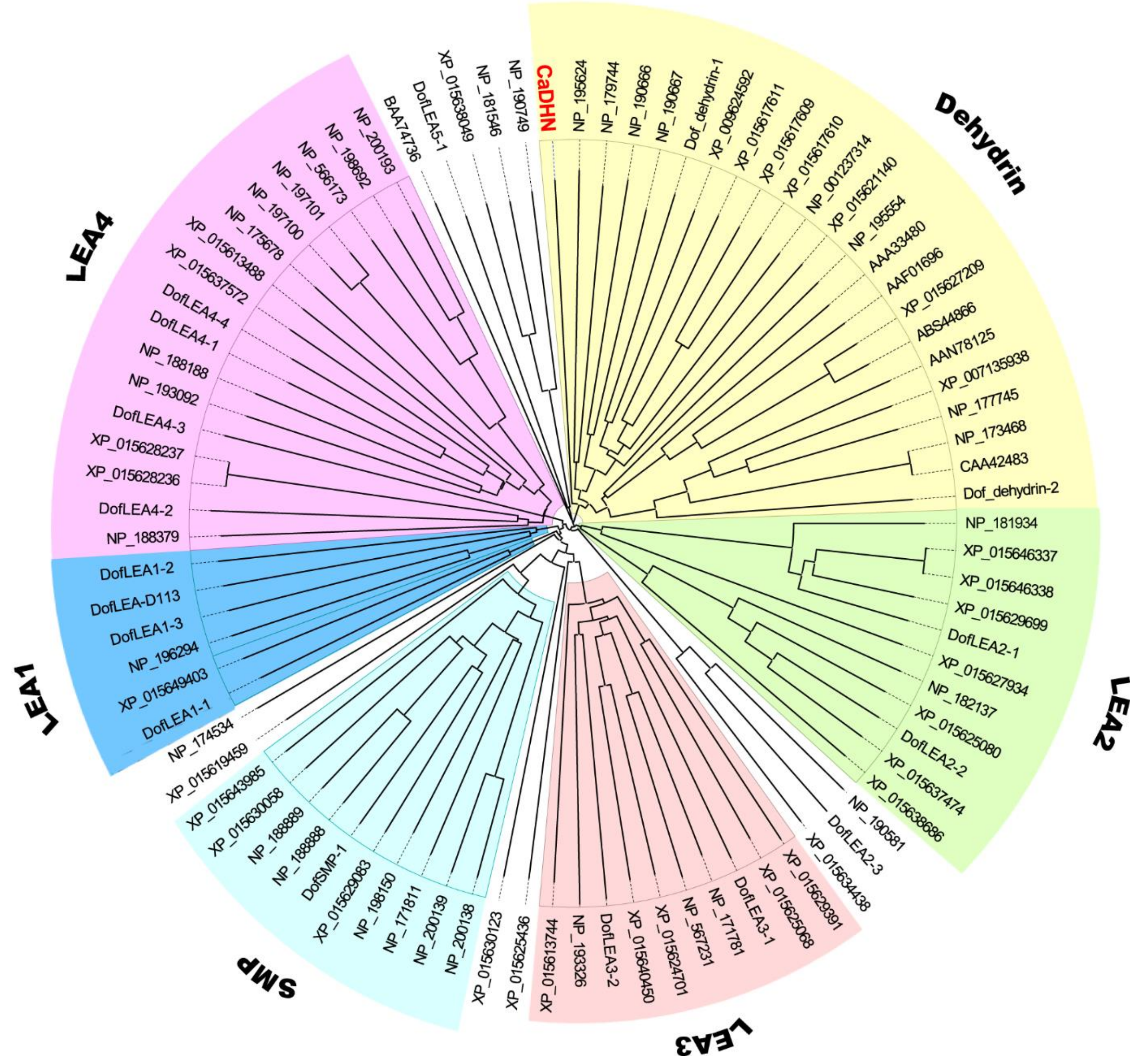

Figure S1. Phylogenetic tree of dehydrins (DHNs) and late embryogenesis-abundant (LEA) proteins. *Cerastium arcticum* DHN (CaDHN), used in this study, is represented in red to highlight the protein. DHN, LEA1, LEA2, LEA3, LEA4, and seed maturation protein (SMP) groups are presented in yellow, blue, green, pale pink, pink, and pale blue, respectively. LEA or DHN proteins that are not yet been comprehensively identified are represented in white, even though they most probably belong to the LEA group. Plants have developed various active defense systems that allow them to resist abiotic stresses, including water loss and drought. Based on these mechanisms, LEA proteins have been widely assigned to three major groups associated with their taxonomic origins (i.e., plants, bacteria, and vertebrates), while other classifications yield five or seven major groups, with nine to fourteen LEA subgroups. DHNs are group 2 members of the LEA protein family, and are present in tissues of different plants. The classification depends on the transcriptional analysis, amino acid

sequences and conserved sequence motifs, three-dimensional protein structures, or chemical features. The phylogenetic tree was generated using *Clustal Omega* and visualized using *FigTree* (http://tree.bio.ed.ac.uk/software/figtree/). Sequences in the tree are indicated using GenBank accession numbers.

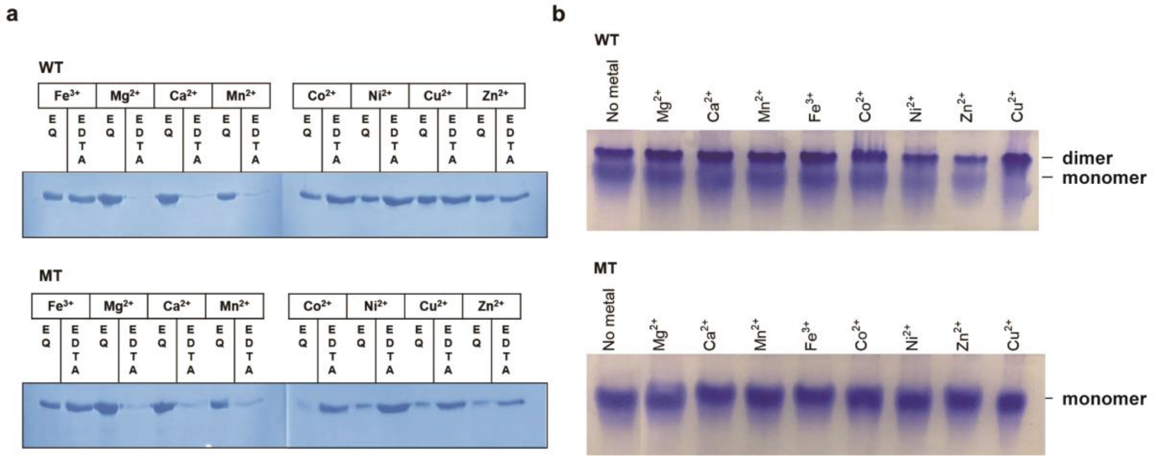

Figure S2. Investigation of metal binding activity (MBA) of *Cerastium arcticum* Lange DHN (CaDHN) using metal ion affinity chromatography (**a**) and native gel analysis; (**b**) The overloaded and unbound proteins were washed with equilibration (EQ) buffer and the metal ion-bound protein was eluted with ethylene-diamine-tetraacetic acid (EDTA). The resulting samples were subjected to SDS-PAGE analysis. Native gel analyses of the CaDHNs was performed in the presence of various metal ions. Note that only the addition of Cu^{2+} ions to the wild type (WT) protein showed position shift of the protein band.

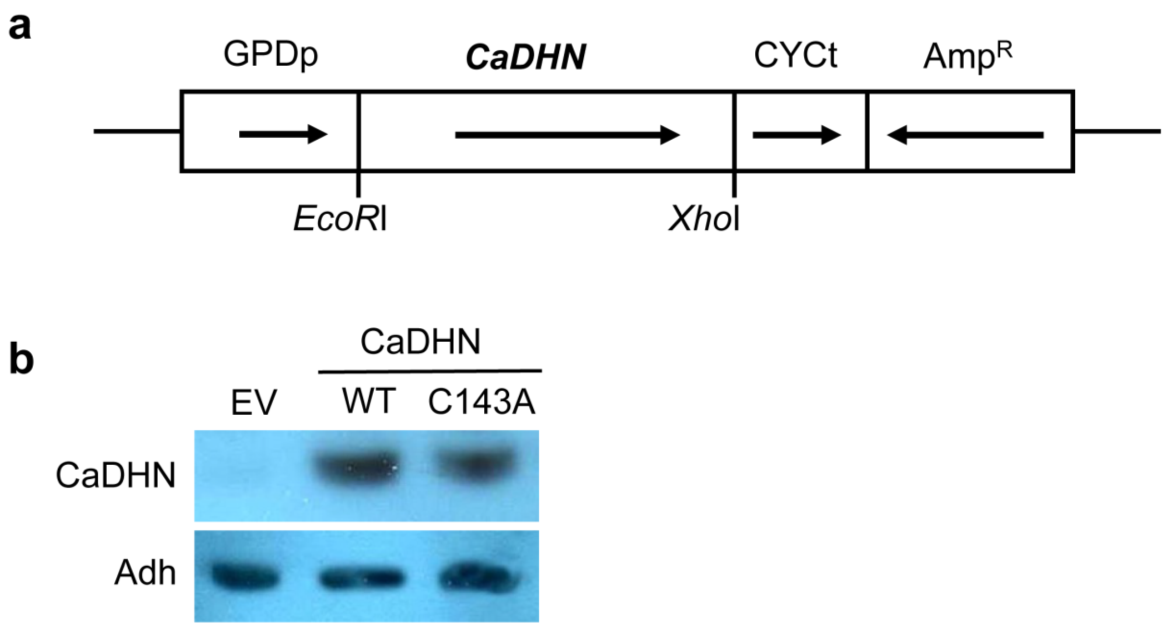

Figure S3. Construction and expression of the *Cerastium arcticum* Lange DHN (CaDHN). (**a**) A portion of the plasmid p426GPD with *CaDHN* gene is shown as a schematic. (**b**) *CaDHN* expression in transgenic yeast cells, as detected by western blot analysis, using Adh antibody from *Saccharomyces cerevisiae* as a loading control. Yeast cells were cultured in YPD media. Crude proteins were extracted from yeast cells in the mid-log phase (A600 = 4.0; OD = 0.6), or over time using glass beads in a lysis buffer containing 20 mM HEPES, pH 7.4; 5 % glycerol; 1 mM dithiothreitol (DTT); 1 mM phenylmethylsulfonyl fluoride (PMSF); and ethylenediaminetetraacetic acid (EDTA)-free protease inhibitor cocktails (Roche Applied Science, Indianapolis, USA). Next, 20 μg of protein was subjected to 15% SDS-PAGE. After electrophoresis, the proteins were electrophoretically transferred to polyvinyl difluoride membranes (Bio-Rad, Hercules, USA) at 180 mA for 3 h at 4 °C. Anti-CaDHN antibodies were diluted in the ratio 1:1000 with blocking buffer. Polyclonal antisera against CaDHN were raised in rabbits (Ab Frontier, Seoul, South Korea). Antibody binding was visualized using enhanced chemiluminescence western blotting detection reagent (GE Healthcare, TX, USA).

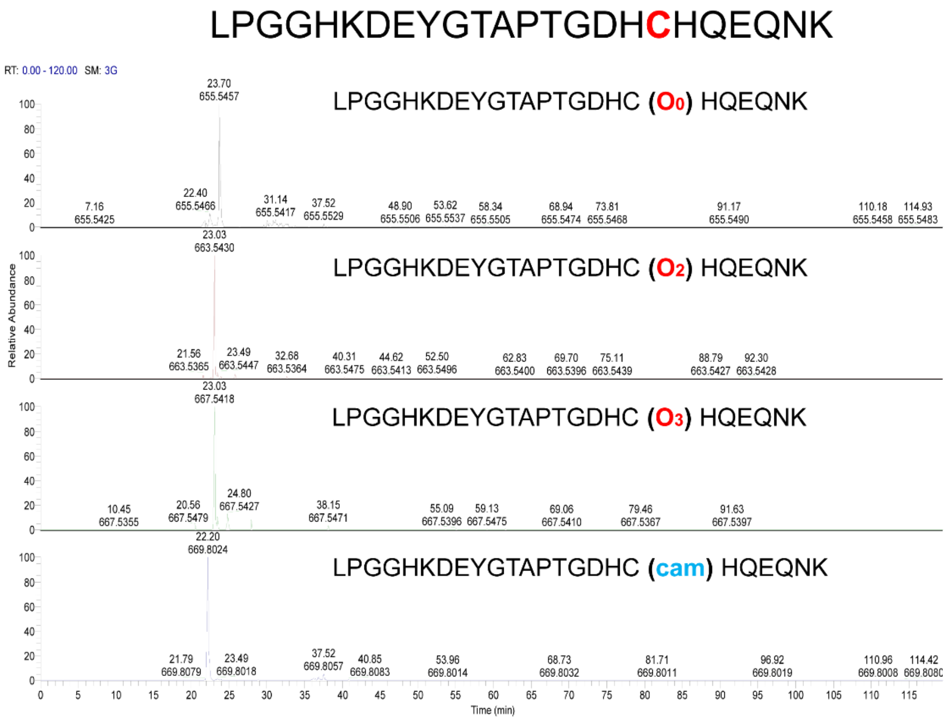

Figure S4. Identification of the cysteine modification using a liquid chromatography tandem mass spectrometer (LC-MS/MS) with a Surveyor nano-flow system, connected to a 7-Tesla Finnigan LTQ-FT mass spectrometer.

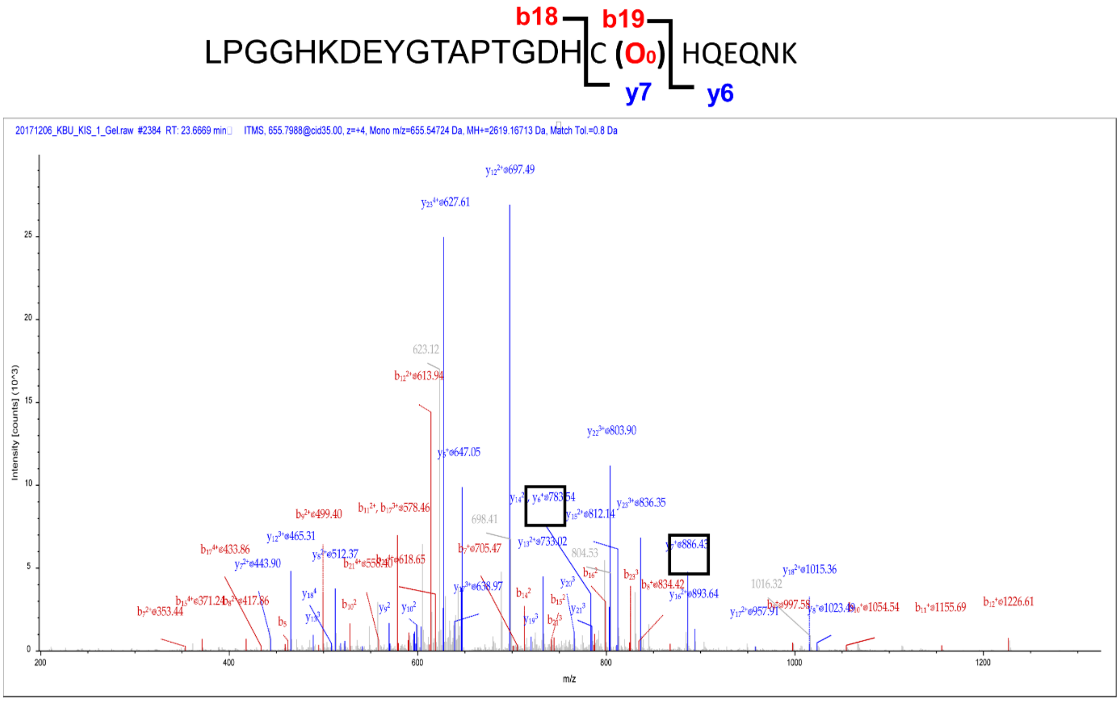

Figure S5. Liquid chromatography tandem mass spectrometer (LC-MS/MS) spectra of the *Cerastium arcticum* Lange dehydrin (CaDHN) peptide containing the cysteine sulfenic acid (-SOH).

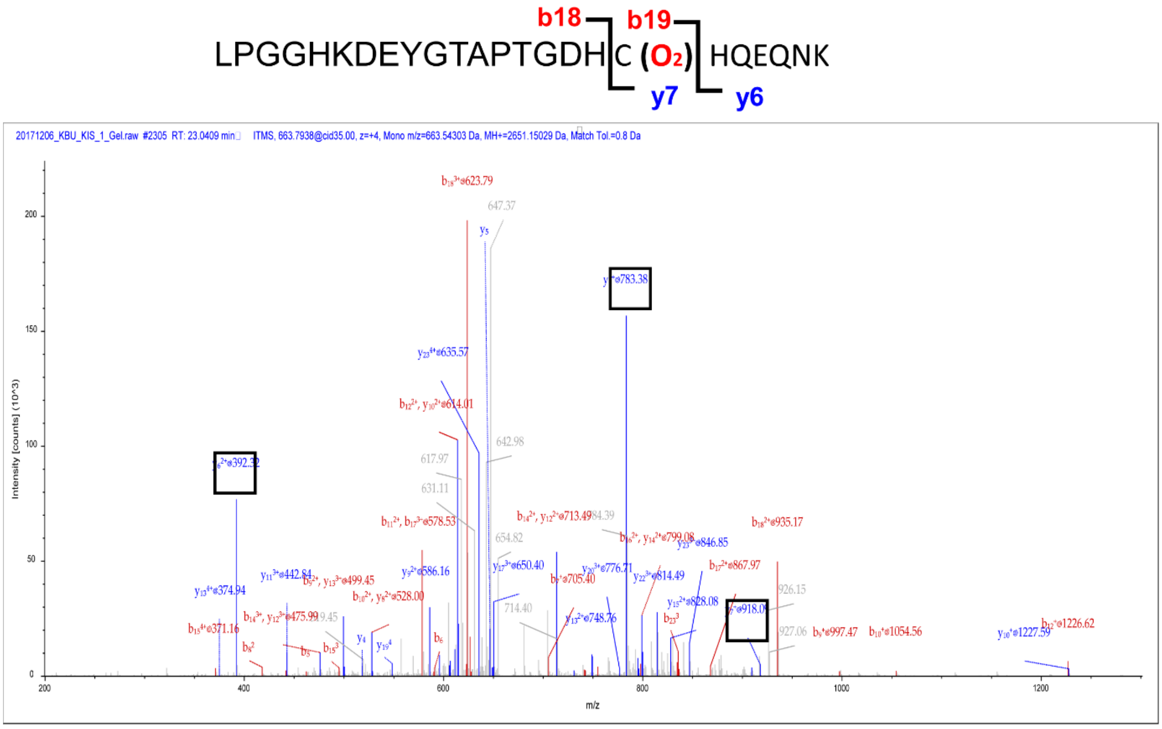

Figure S6. Liquid chromatography tandem mass spectrometer (LC-MS/MS) spectra of the *Cerastium arcticum* Lange dehydrin (CaDHN) peptide containing the cysteine sulfinic acid (-SO_2H).

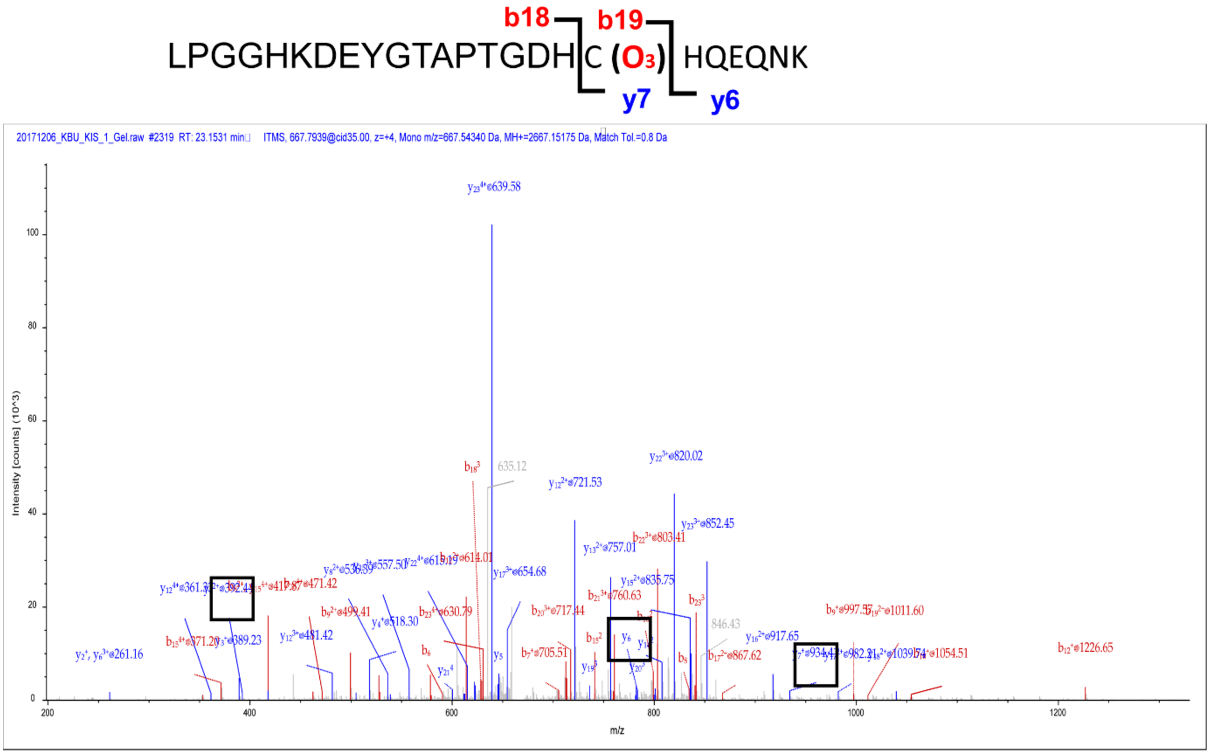

Figure S7. Liquid chromatography tandem mass spectrometer (LC-MS/MS) spectra of the *Cerastium arcticum* Lange dehydrin (CaDHN) peptide containing the cysteine sulfonic acid (-SO_3H).

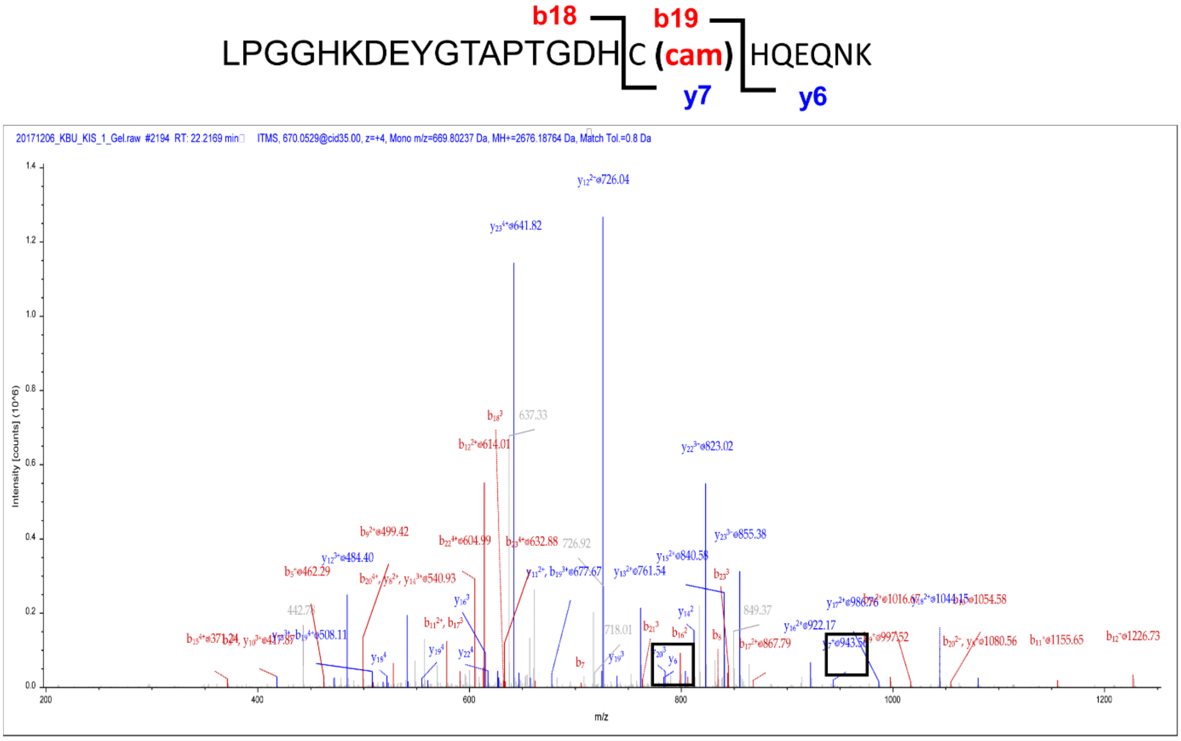

Figure S8. Liquid chromatography tandem mass spectrometer (LC-MS/MS) spectra of the *Cerastium arcticum* Lange dehydrin (CaDHN) peptide containing the carbamidomethyl cysteine (cam).

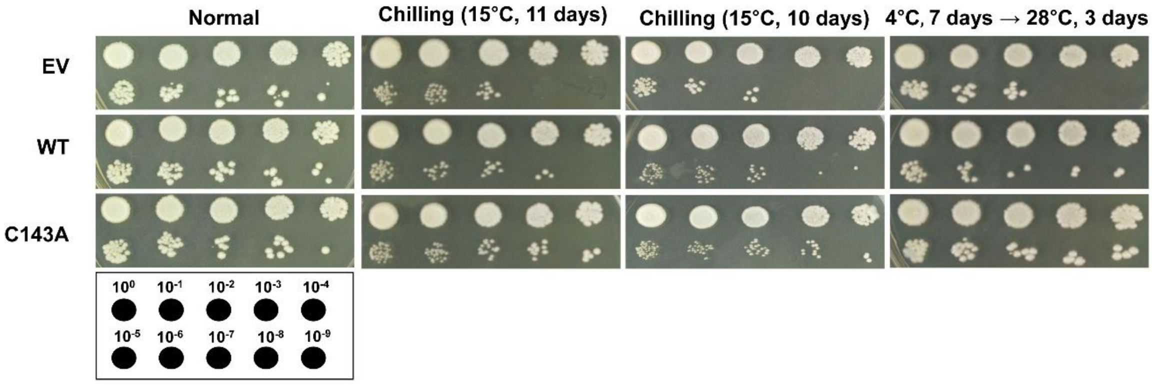

Figure S9. Cell viability of transgenic yeast against thermal stress. The cells transformed with an empty vector (EV) were used as a control. The spotting assay represents at least three independent experiments.

Methods

Metal Binding Activity

The metal affinity column (GE healthcare, TX, USA) was charged with 100 mM metals (Fe^{3+}, Mg^{2+}, Ca^{2+}, Mn^{2+}, Co^{2+}, Ni^{2+}, Cu^{2+}, and Zn^{2+}). Approximately 200 µg of the protein was loaded onto the column that was equilibrated using equilibration (EQ) buffer (20 mM Tris-HCl at pH 8.0 and 200 mM NaCl). The unbound protein on the column was washed out with EQ buffer, and bound protein was eluted with 100 mM EDTA. The eluted samples were collected, subjected to SDS-PAGE, and stained with Coomassie Brilliant Blue R-250.

Western Blot Analysis

Yeast cells were cultured in YPD media. Crude proteins were extracted from yeast cells in the mid-log phase (A600 = 4.0; OD = 0.6) or over time using glass beads in a lysis buffer containing 20 mM HEPES, pH 7.4; 5 % glycerol; 1 mM dithiothreitol (DTT); 1 mM phenylmethylsulfonyl fluoride (PMSF); and ethylenediaminetetraacetic acid (EDTA)-free protease inhibitor cocktails (Roche Applied Science, Indianapolis, USA). Next, 20 µg of protein was subjected to 15 % SDS-PAGE. After electrophoresis, the proteins were electrophoretically transferred to polyvinyl difluoride membranes (Bio-Rad, Hercules, USA) at 180 mA for 3 h at 4 °C. Anti-CaDHN antibodies were diluted to 1:1,000 with blocking buffer. Polyclonal antisera against CaDHN were raised in rabbits (Ab Frontier, Seoul, Korea). Antibody binding was visualized using enhanced chemiluminescence western blotting detection reagent (GE Healthcare).

Cysteine modification

The intact mass was measured by direct infusion into a liquid chromatography tandem mass spectrometer (LC-MS/MS) using a Surveyor nano-flow system, connected to a 7-Tesla Finnigan LTQ-FT mass spectrometer (ThermoFisher Scientific, MA, USA), equipped with a nano-electrospray ion source at the Korea Basic Science Institute (Cheongju, South Korea). The mass spectra were deconvoluted using the ProMass Deconvolution software (ThermoFisher Scientific). To identify the modification of the cysteine residue, the wild type protein was oxidized with H_2O_2 during *Escherichia coli* cultivation. Single polymerization band, visualized on SDS-PAGE, was cut and all free thiols were blocked with excess iodoacetamide for 10 min before tryptic digestion. The peptides were analyzed by LC-MS/MS. The analytes were dissolved in acetonitrile/water (50:50, v/v) and loaded into the pre-opened "medium" borosilicate spray capillaries for off-line nanoelectrospray. A potential of 1000 V was applied to the loaded glass capillary tip. Electrospray ionization mass spectra were collected for 1 min, and the protein masses were calculated by deconvolving multiple charged ions with m/z values and corresponding total charges using the PERCOLATOR program within the Proteome Discoverer software (Thermo

Fischer Scientific), and then manually validated. The considered dynamic modifications on cysteine residues were +15.995 Da for oxidation, +31.990 Da for dioxidation, +47.985 Da for trioxidation, and +57.021 Da for carbamidomethylation.

References

1. Hara, M.; Shinoda, Y.; Tanaka, Y.; Kuboi, T. DNA binding of citrus dehydrin promoted by zinc ion. *Plant Cell Environ*. **2009**, *32*, 532–541, doi: 10.1111/j.1365-3040.2009.01947.x; PubMed PMID: ISI:000265015100009.
